# COVID-19 vaccine barriers among pregnant and lactating refugee women: a case study

**DOI:** 10.3389/fpubh.2025.1600107

**Published:** 2025-07-08

**Authors:** Elisabeth Williams, Ehiremen Azugbene, Fernanda Lozano, Li Liu, Tatiana Patton, Jeanne Nizigiyimana, Crista Johnson-Agbakwu, Alexis M. Koskan

**Affiliations:** ^1^College of Health Solutions, Arizona State University, Phoenix, AZ, United States; ^2^Biodesign Institute, College of Health Solutions, Arizona State University, Tempe, AZ, United States; ^3^Refugee Women's Health Clinic, Valleywise Hospital, Phoenix, AZ, United States; ^4^Collaborative in Health Equity, UMass Chan Medical, Worcester, MA, United States

**Keywords:** COVID-19 vaccine, vaccine hesitancy, refugee, pregnant, lactating, qualitative

## Abstract

**Introduction:**

Pregnant and lactating refugee women rank among the groups least likely to vaccinate against COVID-19. This qualitative study explores their reasons for COVID-19 vaccine hesitancy.

**Methods:**

Between June 2023 and January 2024, cultural health navigators (CHNs) employed by one hospital system conducted in- depth interviews with COVID-19 vaccine-hesitant pregnant and lactating refugee women from five language groups (Arabic, Burmese, Kinyarwanda, Somali, and Swahili). The team also conducted in-depth interviews and a focus group with the five CHNs to further understand community-level factors influencing refugee women's vaccine hesitancy. All qualitative data were analyzed using inductive thematic analysis.

**Results:**

Participants expressed fear of long-term health effects, especially of becoming infertile or of their babies dying, as the primary reasons for not vaccinating. Others reported their perceptions that COVID-19 is no longer a significant health concern. CHNs described the role of social media in spreading misinformation about the vaccine, leading to vaccine hesitancy. Some unanticipated themes that emerged included the role of men in vaccine decision-making and the fear of disrespecting their healthcare provider by declining the vaccine.

**Discussion:**

Study results indicated the need to continue to combat misinformation about the COVID-19 vaccine amongst pregnant and lactating refugee women and the need to take a community-based approach to increase vaccine trust. For example, community health workers or CHNs can provide patient education to increase vaccine trust. Trusted civil organizations could disseminate messages targeting vaccine misinformation spread on social media platforms. Additionally, digital storytelling in refugees' native languages can be a helpful dissemination tool to increase vaccine education and combat misinformation and vaccine hesitancy.

## 1 Introduction

COVID-19 poses significant threats of severe obstetric morbidity and mortality for pregnant and lactating (P/L) women, leading to maternal death, preterm birth, and fetal and infant demise (1, 2). Pregnant women are at considerably higher risk than non-pregnant women for severe complications with COVID-19 (3). These complications have led to increased intensive care unit admissions, tracheal intubation (invasive ventilation), and death (3, 4). Among pregnant women with COVID-19, perinatal outcomes include preterm births and neonatal intensive care unit (NICU) admission (3–5), which can also increase infants' risk for future neurodevelopmental disorders, chronic health conditions, and psychiatric disorders (6, 7). The burden of COVID-19 was grievous in the refugee community due to a host of socioeconomic, environmental, linguistic, and sociodemographic barriers, alongside underlying health morbidity, creating a COVID-19 syndemic, which exacerbated health inequities for this vulnerable population (8).

COVID-19 vaccination helps reduce health complications due to viral infection. Further, the Pfizer BNT162b2 mRNA COVID-19 vaccine is as effective in pregnant women as in the general population, and it is 96% effective in preventing infection and 97% against symptomatic infection after two-dose completion (9). Additionally, when the COVID-19 Omicron variant was circulating, COVID-19 vaccine effectiveness against severe complications in pregnant women was shown to be 74% after the first dose and 91% after the second dose (10). The Centers for Disease Control and Prevention (CDC) reported that mRNA COVID-19 vaccines are safe for pregnant women to receive (11) and strongly recommended P/L women as a priority population for vaccination beginning early in 2021 (12). Ensuring this vulnerable population is vaccinated against COVID-19 is critical to reducing the risk of complications for mothers and children (13).

P/L refugee women face unique challenges accessing and navigating the healthcare system amidst language, literacy, and communication barriers alongside other social determinants of health (e.g., culture, religion) that influence their health and vaccine-related decisions (14). They may face similar barriers as other immigrant and refugee populations, such as language barriers in healthcare, lack of access to reliable information about the vaccine, fear that vaccination could lead to deportation, and systemic barriers such as lack of transportation and computer literacy skills needed to register for vaccination (15, 16) and additional, unexplored barriers related to their current or recent pregnancy. Their barriers and facilitators to vaccinating against COVID-19 may be heterogeneous and distinct from other immigrant and refugee populations, making the current one-size-fits-all vaccination campaigns inadequate. To begin developing precision population health efforts to enhance vaccine campaign effectiveness, it is critical, to identify this distinct populations' barriers to COVID-19 vaccination, including their particular reasons for vaccine hesitancy. To further gain insight into reasons for COVID-19 vaccine hesitancy among the P/L refugee women, we explored the perspectives of cultural health navigators (CHNs), individuals who work with refugee populations and serve as their language and cultural brokers and healthcare system navigators. Therefore, this case study aimed to explore multiple perspectives, including pregnant and lactating refugee women and their CHNs, to identify the various reasons these women experienced COVID-19 vaccine hesitancy.

## 2 Materials and methods

### 2.1 Study design

This case study used a qualitative research design. The study CHNs conducted semi-structured interviews with refugee women who were P/L when they declined the COVID-19 vaccine. The research team conducted individual in-depth interviews and a focus group with the CHNs who served the refugee patients participating in the study.

### 2.2 Setting

Our study worked with one public safety net healthcare system for diverse communities in Arizona, a state that consistently ranks among the top U.S. states welcoming newly arrived refugee populations. The safety net healthcare system is the only public teaching hospital in Arizona, and it provides primary and specialty care services predominantly to underserved, low-income, and ethnically diverse populations, including the growing refugee community. Specifically, we worked with the hospital's integrated women's health clinic, which primarily focuses on refugee patients. This department offers reproductive and preventive health services to the growing refugee community. Medical services provided at this clinic include obstetric, gynecologic, and preventive health care, as well as family planning, and reconstructive procedures for women who endured female genital mutilation/cutting. The patient population of this clinic is diverse and includes more than 9,000 women from 64 countries across Sub-Saharan Africa, Southeast Asia, and the Middle East. To serve the refugee populations, this hospital clinic employs multilingual CHNs from various cultural and linguistic backgrounds to serve as interpreters and health navigators for their patients. Before serving as a CHN, these health workers complete a medical interpreter certification and receive additional on-the-job training to prepare them for their primary clinical responsibilities, including medical interpretation, patient health education, and assistance scheduling appointments. The five CHNs who participated in this study represent the following languages: Arabic, Burmese, Kinyarwanda, Somali, and Swahili. These language groups also represent the majority of refugee patients served by the hospital. The five CHNs served as the face of the study, interviewing patients in their native languages. Two study authors (CJ-A, JN) were co-founders of the hospital's Refugee Women's Health Clinic and had years of experience working with the CHNs, and they introduced the CHNs to the study team. Of note, before the beginning of this research, the study's CHNs provided educational outreach on COVID-19 and COVID-19 vaccines to refugee patients and their families.

### 2.3 Population and sample

To explore P/L refugee women's reasons for avoiding COVID-19 vaccination, the team conducted qualitative research with two groups: P/L refugee women who declined COVID-19 vaccines during the pandemic and CHNs employed by the hospital's women's clinic. This study was part of a larger study investigating COVID vaccine reception by P/L refugee women to create precision public health strategies to increase vaccination in P/L refugee women. In the larger study, the CHNs interviewed 45 women, 30 of whom received COVID-19 vaccines while pregnant or lactating (15). This manuscript focuses on vaccine hesitancy and analyzes data from the 15 P/L women (of the 45 patients interviewed) who refused the vaccine. Thus, the sample size was determined by this predetermined number of women. The last author also interviewed and conducted one focus group with the five CHNs, also refugees, who had unique insights into healthcare decision-making within their respective communities. This information was valuable, particularly as many patients provided limited responses for why they did not want to vaccinate against COVID-19.

The sample of P/L refugee women were purposefully selected from the five language groups previously listed. To participate in the study, the P/L women met the following inclusion criteria: over age 18, currently pregnant or pregnant as early as March 20, 2020 (women in this population typically lactate for up to 2 years or more), never received any COVID-19 vaccines, and willing to provide informed consent. To identify potential participants, the hospital provided a list of patient codes (replacing identifiable information) in the languages spoken by the CHNs. Only hospital staff, including CHNs, could use patient codes to identify potential interview participants. CHNs then called patients to explain the study and inclusion criteria and, among those eligible to participate, to invite them to take part in a one-time, hour-long, in-depth interview. Interviews were scheduled at a mutually convenient time for CHNs and patients and occurred in patients' homes and the hospital.

### 2.4 Data collection

Valleywise Health Medical Center acted as the Institutional Review Board (IRB) of Record (Protocol #2002-072) for this study, and the hospital IRB determined this study was exempt from IRB review.

To ensure the linguistic comfort of the P/L refugee participants, the research team trained the five CHNs, all certified in research ethics before the study, to conduct in-person semi-structured in-depth interviews with the refugee patients in their preferred language. Two study authors, with research experience in working with P/L refugee women and vaccine hesitancy (AK, CJ-A), led the development of the interview guide. See Appendix 1 for the English-language in-depth interview guide for the P/L participants. They sent the interview guide to a professional translation company to translate it into the five study languages and back-translate it into English. The research team also developed a demographic survey to administer to interview participants and had the same company translate and back-translate these documents. The survey included 40 questions that assessed the following information: age, country of origin, ethnicity, marital status, education level completed, religion, employment status, annual household income, number of people (including children) living in the household, U.S. and state residence (number of years), acculturation (eight questions used in previous research) (17), healthcare access (eight questions), COVID-19 history (four questions), and COVID-19 vaccine history (7 questions). Interview participants completed the surveys before their interview.

In May 2023, two research team members (AK) with qualitative research experience (AK, EA) led the in-depth interview training. The training lasted 4 h and covered the following topics: study overview, a description of qualitative interviews, reviewing the informed consent, administering the demographic survey, conducting the in-depth interviews, and practice administering informed consent and conducting semi-structured interviews. The research team members also asked the CHNs to review the interview guides in English and the interview guide translated into their native language to ensure the translations were clear and accurate. Before interviewing began, P/L participants provided verbal consent to participate in the study. The CHNs verbally administered the survey to the participants and conducted all interviews in person with the P/L refugee women between June 2023 and January 2024. P/L interview participants were compensated with a $25 gift card to Walmart for their time.

After CHNs began interviewing patients, to enhance the quality of the interviews, one research team member (EA) reviewed each CHNs' initial interview transcripts and discussed with them strategies to enhance both administering the interview guide and asking probes to clarify responses and encourage more detailed responses. No follow-up interviews were conducted.

To explore the initial P/L refugee community reactions to COVID-19 vaccines during their early roll-out period and better understand the cultural context of the local refugee community, the CHNs also participated in the study as stakeholder participants through both semi-structured interviews and a focus group. The discussion topics for the CHNs included key factors that influenced their community's healthcare decision-making during the COVID-19 pandemic, including social media, specific health beliefs, and community influences that impacted COVID-19 vaccine decisions. The research team, led by the last author, developed the interview guide and conducted in-depth interviews with the CHNs in the study. See Appendix 2 for the interview guide used with the CHNs. Before the CHN interviews and focus groups began, CHNs provided signed consent. The last author also conducted one focus group with the study CHNs to further explore their experiences working on the study. (See Appendix 3 for the focus group guide with CHNs.) For their role as interview participants, the CHNs were compensated with a $100 e-gift card; they were provided a catered meal for their time as focus group participants.

### 2.5 Data analysis

Because of the multilingual nature of the data, a professional transcription and translation company (CommBridge Translations) was utilized to transcribe, translate, and back-translate the interviews. Using an inductive approach, one investigator (AK) read all interview and focus group transcripts and served as the primary codebook creator and editor (18). Research team members conducted a team-based thematic analysis to enhance the rigor of the qualitative analysis to code the interview data (19). The investigator trained the first author in qualitative analysis, and they met biweekly to discuss updates to the codebook and to reach a consensus on coding interview data. They split the transcripts and met, as needed, to discuss and resolve discrepancies in coding, and ultimately agreed on all codes and coded materials before entering coded materials into ATLAS.ti software version 7.0.81. They aggregated the ATLAS.ti output and summarized findings for each code to ensure interpretation consensus before creating a comprehensive summary of the findings (20).

## 3 Results

### 3.1 P/L women demographics

Fifteen refugee women (three per language from the following languages: Kinyarwanda, Arabic, Burmese, Somali, and Swahili) who never received any COVID-19 vaccine doses completed a demographic survey and interview. Participant ages ranged from 23 to 43, with the average age across all linguistic groups 32.9 years. All participants had lived in the U.S. for more than 5 years; across all groups, the average time living in the U.S. was 7.2 years. See Appendix 4 for demographic information of pregnant/lactating refugee patients who participated in the study. All P/L participants were under the primary care of an obstetrician/gynecologist at the time of the interview. All interview guide questions were answered by each P/L participant, and interviews ranged from 2 to 20 min, with a mean of 11 min.

Five CHNs participated in individual interviews and a focus group. All CHNs were female. Their time serving in the CHN role ranged from 4 to 16 years, with an average of 10.3 years. The CHN participants represented diverse backgrounds from Burundi, Iraq, Rwanda, Somalia, and Burma. The CHN interviews lasted between 29 and 60 min, with an average interview time of 45 min, and the focus group lasted 45 min.

### 3.2 Qualitative results

We triangulated data from the P/L refugee patient, CHN interviews, and the CHN focus group. The following themes arose from qualitative data: the evolving perceptions of COVID-19, fears about COVID-19 vaccines, perceptions that the vaccines were ineffective, and the role of misinformation in vaccine decision-making. CHN interviews and focus groups provided richer contextual information for the underlying cultural reasons for vaccine hesitancy, including the role of religion, media, and men in vaccine decision-making and community fears of disrespecting their medical providers.

#### 3.2.1 COVID-19 early and evolving perceptions

Many participants discussed how, initially, they viewed COVID-19 as a serious health issue and vocalized fear of possible COVID-19 infection. The participants' fears about the virus were mainly centered around the fear of death after COVID-19 infection, in large part because of the reports of people dying. Participants reported taking specific steps to avoid infection, such as following stay-at-home orders and wearing masks.

*When COVID started, we were here in Arizona, and when we heard about it, we were scared because many people were dying, many people were getting infected, and we were very scared*. (Participant [P]1067, Kinyarwanda)

However, participants had an evolving view of infection with the virus. As time passed, participants normalized the viral infection and no longer perceived COVID-19 as a significant health risk. They also described their belief that the pandemic was over and that COVID-19 illnesses were now mild.

*In the beginning, when this disease started, it was dangerous, and people were all concerned. But now, after some time has passed, it's become a disease like the flu, meaning it's less severe*. (P1214, Arabic)

They perceived the risk of COVID-19 infection to be less than the risk of vaccination, especially if they became pregnant in the later years of the pandemic. Some participants mentioned that they would be willing to vaccinate if they perceived COVID-19 was becoming a serious health threat again.

*When I got pregnant, COVID seemed to have decreased. People were not afraid anymore; it was very dangerous, though I didn't get infected. Those days were over, and I didn't get infected. I didn't have to take it*. (P1067, Kinyarwanda)

Some communities, particularly the Swahili-speaking community, were skeptical that COVID-19 was an actual illness and believed that COVID-19 was a government-created conspiracy.

*They still don't believe in it because they don't believe in COVID-19 to begin with. They think COVID-19 is a scam; it doesn't exist*. (CHN3)

#### 3.2.2 Fear of receiving COVID-19 vaccines

P/L refugee Women also described numerous fears related to COVID-19 vaccine effects, including becoming infertile, dying after receiving the vaccine, experiencing long-term side effects, and giving birth to babies with severe abnormalities (due to vaccination). Fear of losing their fertility was one of the primary motivators for African participants to avoid the COVID-19 vaccine. They specifically mentioned their fear that receiving the COVID-19 vaccine would negatively impact their menstrual cycle and hormones. One participant attributed her inability to get pregnant to her husband taking the vaccine but later decided that the fertility loss from the vaccine was a rumor.

*I heard that the vaccine would stop the menstruation period and that it would affect the hormones, so that is why I refused to be vaccinated*. (P1121, Somali)

Another noted,

*There was a time when they said if you get it, you won't be able to conceive again*. (P1108, Kinyarwanda)

Despite acknowledging that COVID-19 infection often caused death, several participants stated their rationale for declining the COVID-19 vaccine was their fear that the vaccine caused death.

*I also heard that people died after they got the vaccine, so I tried not to get it. I came from Malaysia, and they said that some pregnant women died after getting the COVID-19 vaccine. So, I did not get the vaccine once I was pregnant*. (P1064, Burmese)

Another motivating reason for avoiding the COVID-19 vaccine was fear of fetal birth abnormalities or the birth of an unhealthy baby.

*I was happy not to get vaccinated. I thought that if I got vaccinated, my unborn child would be abnormal or stillborn*. (P1120, Somali)

P/L participants identified the newness of the vaccine as a reason for fear of congenital abnormalities or stillbirth as a pregnancy outcome. P/L participants did not mention specific birth defects but spoke generically about the possible harm to the fetus because of taking the vaccine. Only one P/L participant stated they knew someone who had a poor pregnancy outcome, which this person associated with the decision to vaccinate against COVID-19.

P/L refugee women witnessed family and friends experiencing side effects from the COVID-19 vaccine and reported that this fear of side effects influenced their decisions to avoid vaccinating.

*My husband had a severe fever after getting the vaccine, and I am afraid that the same would happen to me, that is why I didn't get it*. (P1157, Burmese)

#### 3.2.3 General disinterest in the COVID-19 vaccine

Other participants stated they refused the vaccine simply because they were uninterested in receiving it. Most said no obstacles prevented them from getting the vaccine; they simply did not want it.

*I am not interested in getting vaccinated against COVID-19*. (P1063, Swahili)

Two P/L participants mentioned they did not like needles or shots in general and heard the pain was worse with the COVID-19 vaccine compared to other types of routine vaccinations, keeping them from seeking the vaccine.

Some P/L participants described not needing the vaccine since they used multiple other strategies to keep themselves safe from COVID-19 infection. Specific actions they took to protect themselves and their families included changing their diet by avoiding junk food and sweets, increasing their intake of fruits and vegetables, ensuring cleanliness at home, hand washing/sanitizing, masking when at work and school, avoiding crowded areas, avoiding infected people, and staying home. P/L participants who were unemployed in the formal sector also perceived they had a lower risk of contracting the virus due to reduced exposure to other adults. Therefore, they perceived that they did not need the vaccine.

*I'm not allowed to eat chips or sweets because I know the virus thrives on sugary food. So, my main concern was to protect myself and my family. I started eating healthy food, like lots of vitamin C, oranges, fruits, vitamins, and soups. I also took vitamin D and vitamin C supplements. The same goes for my children. I made sure they ate healthy food and completely avoided junk food and sweets*. (P1045, Arabic)

Another P/L participant noted,

*I believe that if you keep good hygiene and stay away from people who are infected, you will be safe*. (P1121, Somali)

#### 3.2.4 Questioning COVID-19 vaccine effectiveness

Many P/L participants were aware of COVID-19 variants and viral mutations and questioned its impact on vaccine effectiveness, leading them to avoid vaccination. They believed the need for additional booster doses indicated the vaccine's ineffectiveness. Several participants noted they witnessed family and friends get vaccinated but eventually contracted COVID-19, illustrating the vaccines' ineffectiveness, another reason they did not want to be vaccinated.

*I knew that COVID keeps mutating, meaning it's not the same virus every time; it keeps changing. So, my belief was that the vaccine wouldn't be effective for the different variants of COVID*. (P1045, Arabic)

#### 3.2.5 COVID-19 vaccine misinformation

Discussions with the CHNs helped provide additional context and background for the P/L participant interviews, as the research team was able to discuss the role of misinformation on refugee women's vaccine hesitancy and explore cultural factors that influenced their vaccine decision-making. Both traditional and social media played a significant role in vaccine decision-making in refugee communities. However, the CHN interviews confirmed that most participants engaged with foreign news media and social media apps that cater to an international audience (WhatsApp and Facebook).

*But I will say 80% of our communication with our community members, whether in the States or abroad, is on WhatsApp…*

*WhatsApp is so important for us because they can use it wherever they have Wi-Fi*. (CHN5) Another CHN noted,

*We have it [COVID-19 information] in our own language, we can all view in the news, the media, the social media. So, they listen from there. The news, all the updated news. Like, here it's like Fox News*. (CHN1)

Both P/L participants and CHNs discussed how rumors, fears, and misinformation circulated through the refugee communities. Refugee communities' vaccine-related fears included the following: they cause infertility, contain microchips, were lab-created to bring the end of the world, and China deliberately shipped ineffective or tainted vaccines to their countries. These rumors were spread by social media, primarily through Facebook, WhatsApp, YouTube, and Instagram.

Some participants reported seeing messages by religious leaders from their home countries spreading rumors about the vaccines.

*The most significant thing is on social media, where some people talk about the vaccine not being good and causing many illnesses, which made me hesitant*. (P1214, Arabic)

Another refugee patient noted,

*We used to find groups on WhatsApp, and you find people who post videos like this, and they say scary things about COVID… They were mocking the vaccines and even talking about scary things. That is what made me avoid it and feel that maybe if I give it, I will not be able to get pregnant again*. (P1067, Kinyarwandan)

P/L participants noticed that the messaging they saw on social media included both positive and negative things about the COVID-19 vaccine, which one participant identified as contributing to the confusion and fear around the vaccine. P/L participants had difficulty parsing out truth/fact vs. rumors/fiction. No one identified the sources specifically making these videos–just vague “people” and “they.”

*I've seen people positively advocating for the vaccine, and at the same time, others criticize it negatively. So, it creates fear in people. … The most significant thing is on social media, where some people talk about the vaccine not being good and causing many illnesses, which made me hesitant*. (P1214, Arabic)

#### 3.2.6 Political and religious influences on COVID-19 vaccine uptake

The political climate in the U.S. during the COVID pandemic did not impact vaccine decision-making for P/L participants, as no one cited politics as a direct factor for their vaccine hesitancy. Some P/L participants noted that others they knew had been vaccinated as new refugee arrivals, to obtain their permanent resident status. The CHNs noted refugee communities' fear of deportation if people did not take the COVID-19 vaccine. The CHNs mentioned that the Swahili-speaking community perpetuated this fear of deportation, primarily through religious leaders and pastors, including religious leaders currently residing overseas.

Overseas religious leaders played a significant role in perpetuating rumors and fears of the vaccine. Several CHN participants mentioned their communities often watched religious services overseas on Zoom or received videos of overseas religious leaders through WhatsApp, cautioning their audience to avoid the COVID-19 vaccines because of fears of experimenting on the population or tracking devices that could be implanted in those that received the vaccine.

However, CHNs did state that outside of Jehovah's Witnesses, no refugee religions expressly prohibited vaccinations or treatment for disease as part of the tenets of that religion. Thus, religion and religious rules did not appear to play a large factor in vaccine hesitancy for refugee women. However, individual trusted religious leaders, often ones located overseas in Africa, preached against the vaccine, leading to vaccine hesitancy in the refugee communities in Phoenix.

*Yeah, because when you said, “Your pastor is out there in Africa. He's not here and doesn't know what's going on here. So, do you really still believe him?” they tell you, they say, “I have been with that pastor since I was a teenager. Why would I trust what I hear from people here more than my own pastor?”* (CHN3)

#### 3.2.7 Cultural influences on vaccine hesitancy

CHNs described other cultural factors that seemed to play a role in COVID-19 vaccine hesitancy for refugee women.

One CHN mentioned that, as a group, her community tended to avoid injections as much as possible, preferring to receive medications orally rather than through a needle, in part because of fear of the pain of injection but also fear of what might be in a syringe.

*As a medical interpreter, I get to know them very well. They do not like injections. I don't like injections. I don't like needles at all. So, if you put a Tylenol, liquid Tylenol in a syringe with a needle, and they put the Tylenol–the pill, next to each other, and you say, which one can I give you? They both are Tylenol. Which one can I give you? In the needle, it works faster. It'll go through your veins and the blood, and you'll feel better faster. They will say no. No, I don't want the injection. I will take the pill*. (CHN2)

Several CHNs also described how the idea of fatalism impacted their communities' healthcare decision-making, including COVID-19 vaccine decision-making. Some refugee communities were more accepting of poor health outcomes because of the belief that God predetermined the outcome and would have occurred regardless of medical intervention.

*And God knows the best, yeah, but the good thing is we Muslims, as believers, think everything was written and was written for us to happen. So, regardless of whether it's COVID or not, it was written. And she said that. “It was written that I would lose these pregnancies*. (CHN2)

The CHNs also identified that some refugee communities lack an overall culture of prevention, potentially influencing vaccine decision-making for participants. CHNs discussed how patients vocalize that they do not need to partake in uncomfortable medical procedures such as vaccinations, mammograms, and cervical screenings since they do not feel anything is wrong. While no participants explicitly discussed this lack of preventive healthcare, this cultural phenomenon may have played an important role in vaccine decision-making during the COVID-19 pandemic.

*As long as they are walking, sleeping, and doing their activities normally, they say, “We are good. There's nothing wrong with me…Even with the well-woman exam, they refuse to do it. “Why should I do it? I'm good.” “I don't have a husband,” one of them said to me, “I haven't had a husband for 13 years. Why do I do it? I'm good. There is nothing wrong with me.” So, yeah, even mammograms, they refuse to do it*. (CHN4)

However, some CHNs noted that their communities' religious beliefs endorsed preventive healthcare and modern medicine as part of religious practice.

*Because Islam encourages medicine, Islam has not forbidden us from seeking vaccinations because Islam encourages that we take care of ourselves*. (CHN2)

#### 3.2.8 Role of men in vaccine decision-making

During the focus group, the CHNs discussed the role of men in healthcare decision-making, including vaccine decision-making, for the women in the family. While many study participants mentioned that their husbands and partners received the vaccine primarily as part of employment requirements, they declined the COVID-19 vaccine, potentially because of the influence of men in their decision-making for their families.

*And also, I mean the decision-making too, like [name] said, a lot of men make decisions for their wives about the vaccine*. (CHN5)

One other CHN discussed the role in women's healthcare decision-making.

*I think it's similar in our communities, the whole community …because even when you are asking the wife, the provider is asking the wife about birth control, she will tell you, “I need to talk to my husband first.”* (CHN3)

Another commented more broadly,

…*Men are the household decision-makers*. (CHN1).

One CHN noted men's roles in their family members' vaccine-related decisions.

*Some of my patients said, “My husband doesn't want me and my kids to take it.” He took it, but he doesn't want us to take it*. (CHN 4)

However, in some communities where women have more formal education and employment, women are more likely to make healthcare decisions outside of the influence of their husbands and partners. For example, one CHN stated,

*I can say that's the truth in our community; that's true of some women. They [women] make their own decisions. For those who are educated and work, they do whatever they want*. (CHN4)

#### 3.2.9 Impact of vaccine hesitancy on patient-provider relationship

During the CHN interviews and focus group discussion, CHNs discussed the refugee patients' fears of how declining the COVID-19 vaccine may impact their relationship with their trusted medical provider. The CHNs describe how, in many refugee countries of origin, the relationship between the medical provider and the patient is often more paternalistic and less focused on shared decision-making, where the provider is the authority, and patients do as they are told. CHNs discussed how clinic patients were concerned that their trusted medical provider might feel disrespected if they declined the COVID-19 vaccination and feared that disrespect would alter their patient-provider relationship dynamic for the worse.

*They asked about the consequences if they refuse and if it will affect the services at the clinic. Would the relationship still be good between me and my provider if I said no? And we have to assure this is definitely not going to affect the relationship between you and your provider; [it's] not going to be against you, no one's going to write up, no one's going to say anything against you, it's just advice and suggestion from your provider for what's good for your health and your baby*. (FG, CHN2)

## 4 Discussion

This study explored pregnant and lactating refugee women's COVID-19 vaccine hesitancy. Other research identifies facilitators of vaccine uptake among this same population (21). Although past research has examined general vaccine hesitancy in refugee and immigrant populations (22), this study adds to the relatively few studies that specifically explore the COVID-19 vaccine perceptions among women who were pregnant or who had recently given birth. Our study offers novel insights into the unique concerns of pregnant and lactating refugee women, particularly as it relates to their profound fears about the COVID-19 vaccine causing birth defects, negatively impacting their babies, and, most notably, causing infertility. Similar to research with refugee and migrant women (but unrelated to vaccine research), fertility was deeply tied to women's identities, social roles, and cultural norms (23). The fear of losing their fertility remained heightened, even among women who already had several children. These concerns highlight the participants' sense of self, their perceived value within their families and communities, and broader cultural expectations emphasizing large families. Although the CDC reassured the public that COVID-19 vaccines are safe for pregnant women and do not affect fertility (2), our refugee patients still feared the vaccine's potential effects. However, refugee communities may not view U.S. government agencies, such as the CDC, as a trusted authority for health information, so utilizing other trusted partners for health messaging may be crucial to reaching this population. To address refugees' vaccine fears, campaign planners should collaborate with community partners and trusted cultural health navigators or community health workers to inform the messaging for vaccine-related outreach to refugee communities (24, 25). In addition, vaccine campaigns should prioritize clear, evidence-based communication about the safety of COVID-19 vaccines, particularly for pregnant and lactating refugee women, to mitigate these fears and improve vaccine uptake in these vulnerable populations.

Another prominent reason for vaccine hesitancy among the study participants was a low perceived need to vaccinate against COVID-19. Many refugee women, particularly those who are stay-at-home parents, believed they were at lower risk of COVID-19 infection due to their limited social interactions and strict adherence to other preventive measures. Some women's husbands received the vaccines to continue working at their jobs. However, because the husbands were vaccinated, they did not believe they also needed the vaccine. This finding aligns with similar studies on other refugee and immigrant populations, who reported a low perceived need for COVID-19 vaccines (22, 24, 26). Also, other U.S. racial and ethnic minority populations have questioned the effectiveness of COVID-19 vaccines' especially given the necessity for additional booster doses (27, 28). COVID-19 vaccine campaigns should focus on highlighting the ongoing risks associated with the virus, particularly for pregnant women, emphasizing that the danger remains significant for vulnerable groups.

Misinformation about COVID-19 vaccines, primarily spread through social media, played a significant role in vaccine decision-making for P/L refugee study participants. Early in the pandemic, there was a lack of clear, authoritative information about the COVID-19 virus as scientists worked to understand the virus. This dearth of information opened an online space for the proliferation of misinformation on digital media platforms, including social media apps (29, 30). Patterns of online search queries in commonly used search engines show that misinformation is linked to the COVID-19 vaccine, as the spread of COVID-19 misinformation peaked after the FDA approved the COVID-19 vaccine and during each successive vaccine announcement (30). By its nature, social media allows for the quick spread of rumors and misinformation (31); indeed, studies have shown that individuals who use social media as an information source are less likely to have correct information than those who receive information from a healthcare provider (32). Prior research has also shown that, overall, many refugee communities exhibit vaccine hesitancy, often compounded by distrust in the health system (20, 33).

Uniquely, study participants noted that the misinformation accessed by refugee communities is often created in other languages and perpetuated by individuals outside of the U.S., complicating the ability of officials and medical providers to combat the misinformation, as they may not be aware of specific overseas rumors. Furthermore, less is known about the use of WhatsApp to circulate health information, especially since the content of WhatsApp is end-to-end encrypted rather than public, as in other social media apps, and thus, is not as available for researchers to study (34).

However, recent studies in Mexico have shown that WhatsApp played a significant role in circulating COVID-19 misinformation (35), leading to questions about the best way for health officials to combat misinformation circulating on social media messaging apps. However, opportunities exist to provide COVID-19 vaccine information through relevant health education. Some studies have shown that using trusted civil organizations to randomly disseminate messages targeting vaccine misinformation using social media, such as WhatsApp, may increase knowledge and encourage behavior change (36). Officials and medical providers should also consider non-Western ways of conveying health information, including using storytelling as a viable method of health communication. Prior research has shown that storytelling as a health communication medium can help combat vaccine hesitancy (34, 37). Persuasive storytelling through firsthand perspectives from trusted community members, such as sharing stories of becoming ill with COVID-19 or COVID-19 vaccination experiences, could be a viable way of combating medical misinformation circulating within refugee communities, as well as an opportunity to build trust in the medical system of the U.S. (34, 37).

While all P/L participants had lived in the U.S. for at least 5 years and received their permanent legal residency status 4 or more years ago, some P/L participants mentioned that others they knew had to receive a COVID-19 vaccination as part of their legal status adjustment process. This legal requirement led to misinformation and fears about deportation, even for those individuals with secured legal status. By law, refugees are required to adjudicate their refugee status to lawful permanent resident status (“green card”) 1 year after arriving in the U.S. Adjudicating status also requires immigrants to comply with U.S. vaccine requirements, which was amended in 2021 to include at least one dose of a COVID-19 vaccine (38). Providing correct information, both medical and legal, is crucial to combat vaccine misinformation for a refugee population. Medical clinicians and CHNs are situated to play an essential role in promoting accurate health information to their refugee patients, both during the initial resettlement period and even into the later years of an individual's resettlement. Basic clinician awareness of how healthcare immigration policies impact patients can help build and enhance a trusting relationship with their clients. Additionally, medical providers who see refugee patients can build relationships with their local refugee resettlement agencies, which are highly trusted by refugees and typically employ culturally and linguistically competent staff to assist refugees upon initial arrival. Refugee resettlement agencies are uniquely situated to offer clinicians and health navigators a point of contact for current immigration policies and can act as a point of trusted health education during global health crises such as the COVID-19 pandemic (39, 40).

While most P/L participants and CHNs endorsed preventive healthcare, some CHNs discussed the role of religious fatalism in guiding health behaviors. Religious fatalism is the belief that disease prevention is outside of individual locus of control and, instead, God or other external forces predetermine health outcomes (41). This finding aligns with prior research on religious fatalism and its impact on refugees' healthcare decision-making (42, 43).

Medical providers should be aware that patients may come from a socio-cultural background of spiritual fatalism, which may influence healthcare decision-making, especially the acceptance of certain preventive medical interventions, including vaccination. Promoting that vaccination can help prevent unnecessary morbidity and mortality, as well as preventing even mild cases of illness, can be a health education strategy that medical providers can utilize with their refugee populations.

Our research found that some pregnant and lactating refugee women included spouses (husbands) in their healthcare decision-making, including vaccination. Past research found that men are typically the central decision-making authority for household healthcare matters, but women are also often involved in this decision-making (44). Other research also supports that joint decision-making between spouses plays a high role in deciding whether to obtain a vaccine, with some reporting that the decision relies entirely on the man (45). In this case, asking one's husband for permission regarding a healthcare decision is often associated with showing respect. Future vaccine-related health campaigns for pregnant/lactating refugee women should include the male perspective and their role in influencing vaccination decision-making. Further insight into the male perspective as it applies to the dynamics of different cultural households and traditional values could help address and understand vaccine hesitancy among refugee populations.

### 4.1 Limitations

The strength of this study was the qualitative case study approach, which employed multiple qualitative methods with two related stakeholder groups. This case study approach allowed for a greater depth of information and experience and provided richer insight into COVID-19 vaccination decision-making for this population in this hospital-based clinic setting. However, this study includes several potential limitations that could impact study findings. While the CHNs received training to conduct interviews in a consistent, reliable, and professional manner (with three of the CHNs having experience in giving interviews in the past), each CHN may have slightly differed in practice when conducting the interviews, including their use of follow-up probes and clarifying questions. For example, one CHN conducted an interview that lasted slightly more than 2 min, reflecting less rich interview data and treating the interview guide as a survey rather than using probes to encourage fuller descriptions, as practiced in the interview training workshop. To enhance the qualitative interviewing and resulting data, one study team member met with all CHNs to discuss enhancing the interviews after their initial interviews. This helped elicit longer, richer conversations. Thus, the results obtained from this study could be different from those of other studies with interviews conducted by one or two qualitative research experts. However, this community-based approach to qualitative interviews encouraged limited English-proficient refugee P/L patients to participate in this study, which is considered a strength of the study. Another limitation includes the small sample size of P/L refugee women in this study. However, the authors agreed that information saturation was reached with a sample size of 15 participants, and no new themes would emerge with a larger sample size. After their interviews, some P/L refugee women reported their fear of being recorded. Such fear may have affected their willingness to answer all interview questions fully. Finally, the refugee women in this study have lived in the U.S. for 4 or more years. Their responses may differ from those of newly resettled refugees. Future studies could consider taking newly resettled refugees as part of the sample to capture these potentially varying responses. Another limitation is the potential bias resulting from two study team members being well-acquainted with the CHNs. However, patient participants remained blinded to all research team members (other than the individual CHNs conducting the interviews), and these two team members did not participate in the data collection, coding, or analysis.

## 5 Conclusions

Vaccine hesitancy among pregnant and lactating refugee women resulted in a lack of COVID-19 vaccine uptake.

Hesitancy was often fueled by fear of adverse reactions to the vaccine, including fear of fertility loss and birth abnormalities. Hesitancy was also informed by misinformation, often spread via social media. Future vaccination campaigns should consider appropriate ways to address vaccine information and misinformation within refugee communities. They should seek to deliver healthcare information in a culturally competent manner, utilizing trusted institutions, such as resettlement agencies and lay health workers. CHNs offer the possibility to build trust with refugee communities and build on existing relationships to address new medical innovations such as vaccines.

## Data Availability

The raw data supporting the conclusions of this article will be made available by the authors, without undue reservation.

## References

[1] Razzaghi H Meghani M Pingali C Crane B Naleway A Weintraub E. COVID-19 vaccination coverage among pregnant women during pregnancy—eight integrated health care organizations, United States, December 14, 2020–May 8, 2021. MMWR Recomm Rep. (2021) 70:895–9. 10.15585/mmwr.mm7024e2 34138834 PMC8220952

[2] Centers for Disease Control and Prevention. COVID-19 Vaccines While Pregnant or Breastfeeding. (2024). Available online at: https://www.cdc.gov/covid/vaccines/pregnant-or-breastfeeding.html?CDC_AAref_Val=https://www.cdc.gov/coronavirus/2019-ncov/vaccines/recommendations/pregnancy.html (accessed September 22, 2021).

[3] Zambrano LD Ellington S Strid P Galang RR Oduyebo T Tong VT. Update: characteristics of symptomatic women of reproductive age with laboratory-confirmed SARS-CoV-2 infection by pregnancy status—United States, January 22–October 3, 2020. MMWR Recomm Rep. (2020) 69:1641–7. 10.15585/mmwr.mm6944e3 33151921 PMC7643892

[4] Mullins E Hudak ML Banerjee J Getzlaff T Townson J Barnette K. Pregnancy and neonatal outcomes of COVID-19: coreporting of common outcomes from PAN-COVID and AAP- SONPM registries. Ultrasound Obstet Gynecol. (2021) 57:573–81. 10.1002/uog.23619 33620113 PMC8014713

[5] Nana M Nelson-Piercy C. COVID-19 in pregnancy. Clin Med. (2021) 21:e446–50. 10.7861/clinmed.2021-0503 34507928 PMC8439502

[6] Crump C. An overview of adult health outcomes after preterm birth. Early Hum Dev. (2020) 150:105187. 10.1016/j.earlhumdev.2020.105187 32948365 PMC7480736

[7] Saigal S Doyle LW. An overview of mortality and sequelae of preterm birth from infancy to adulthood. Lancet. (2008) 371:261–9. 10.1016/S0140-6736(08)60136-1 18207020

[8] Sekalala S Perehudoff K Parker M Forman L Rawson B Smith M. An intersectional human rights approach to prioritising access to COVID-19 vaccines. BMJ Glob Health. (2021) 6:e004462. 10.1136/bmjgh-2020-004462 33627362 PMC7907827

[9] Dagan N Barda N Biron-Shental T Makov-Assif M Key C Kohane IS. Effectiveness of the BNT162b2 mRNA COVID-19 vaccine in pregnancy. Nat Med. (2021) 27:1693–5. 10.1038/s41591-021-01490-8 34493859

[10] Ellington S Jatlaoui TC. COVID-19 vaccination is effective at preventing severe illness and complications during pregnancy. Lancet. (2023) 401:412–3. 10.1016/S0140-6736(22)02613-7 36669521 PMC9844991

[11] Zauche LH Wallace B Smoots AN Olson CK Oduyebo T Kim SY. Receipt of mRNA COVID-19 vaccines preconception and during pregnancy and risk of self-reported spontaneous abortions, CDC v-safe COVID-19 vaccine pregnancy registry 2020-21. Res Sq. (2021). 10.21203/rs.3.rs-798175/v1 34401872 PMC8366802

[12] Grunebaum A Chervenak FA. Physician hesitancy to recommend COVID-19 vaccination in pregnancy as a cause of maternal deaths - Robert Brent was prescient. Birth Defects Res. (2023) 115:1255–60. 10.1002/bdr2.2136 36515139 PMC9878218

[13] Lau LS Rodgers G. Cultural competence in refugee service settings: a scoping review. Health Equity. (2021) 5:124–34. 10.1089/heq.2020.0094 33778315 PMC7990563

[14] Feinberg I O'Connor M Owen-Smith A Dube S. Public health crisis in the refugee community: little change in social determinants of health preserve health disparities. Health Educ Res. (2021) 36:170–7. 10.1093/her/cyab004 33599272 PMC7928937

[15] Koku EF Johnson-Yengbeh N Muhr A. Addressing COVID-19 vaccine hesitancy and uptake among African immigrants: lessons from a community-based outreach program. J Racial Ethn Health Disparities. (2024) 12:1124–38. 10.1007/s40615-024-01947-9 38443740 PMC11913973

[16] McFadden SM Demeke J Dada D Wilton L Wang M Vlahov D. Confidence and hesitancy during the early roll-out of COVID-19 vaccines among black, Hispanic, and undocumented immigrant communities: a review. J Urban Health. (2022) 99:3–14. 10.1007/s11524-021-00588-1 34940933 PMC8697839

[17] Johnson-Agbakwu CE Flynn P Asiedu GB Hedberg E Breitkopf CR. Adaptation of an acculturation scale for African refugee women. J Immigr Minor Health. (2016) 18:252–62. 10.1007/s10903-014-9998-6 24573644 PMC4183716

[18] Kuper A Reeves S Levinson W. An introduction to reading and appraising qualitative research. BMJ. (2008) 337:a288. 10.1136/bmj.a288 18687727

[19] Saldaña J. The Coding Manual for Qualitative Researchers. Thousand Oaks: Sage (2013).

[20] Sutton J Austin Z. Qualitative research: data collection, analysis, and management. Can J Hosp Pharm. (2015) 68:226–31. 10.4212/cjhp.v68i3.1456 26157184 PMC4485510

[21] Azugbene EA Koskan AM Williams E Patton T Liu L Nizigiyimana J. Facilitators of COVID-19 vaccination among pregnant and lactating refugee women: a qualitative study using a community-based approach. Patient Educ Couns. (2025) 136:108778. 10.1016/j.pec.2025.108778 40233600

[22] Daniels D Imdad A Buscemi-Kimmins T Vitale D Rani U Darabaner E. Vaccine hesitancy in the refugee, immigrant, and migrant population in the United States: a systematic review and meta- analysis. Hum Vaccin Immunother. (2022) 18:2131168. 10.1080/21645515.2022.2131168 36332155 PMC9746503

[23] Hawkey AJ Ussher JM Perz J. “If you don't have a baby, you can't be in our culture”: migrant and refugee women's experiences and constructions of fertility and fertility control. Womens Reprod Health. (2018) 5:75–98. 10.1080/23293691.2018.1463728

[24] Aghajafari F Wall L Weightman A Ness A Lake D Anupindi K. COVID-19 vaccinations, trust, and vaccination decisions within the refugee community of Calgary, Canada. Vaccines. (2024) 12:177. 10.3390/vaccines12020177 38400160 PMC10891815

[25] Thomas CM Yun K Mudenge NU Abudiab S de Acosta D Fredkove WM. Experiences of American health departments, health systems, and community organizations in COVID-19 vaccine provision for refugee, immigrant, and migrant communities. Am J Trop Med Hyg. (2023) 109:471–9. 10.4269/ajtmh.23-0034 37429571 PMC10397449

[26] Deal A Crawshaw AF Carter J Knights F Iwami M Darwish M. Defining drivers of under-immunization and vaccine hesitancy in refugee and migrant populations. J Travel Med. (2023) 30:taad084. 10.1093/jtm/taad084 37335192 PMC10481413

[27] Doherty IA Pilkington W Brown L Billings V Hoffler U Paulin L. COVID-19 vaccine hesitancy in underserved communities of North Carolina. PLoS ONE. (2021) 16:e0248542. 10.1371/journal.pone.0248542 34723973 PMC8559933

[28] Razai MS Osama T McKechnie DG Majeed A. COVID-19 vaccine hesitancy among ethnic minority groups. BMJ. (2021) 372:n513. 10.1136/bmj.n513 33637577

[29] Roschke K Koskan AM Sivanandam S Irby J. Partisan media, trust, and media literacy: regression analysis of predictors of COVID-19 knowledge. JMIR Form Res. (2024) 8:e53904. 10.2196/53904 39047283 PMC11306951

[30] Merrick E Weissman JP Patel SJ. Utilizing Google trends to monitor coronavirus vaccine interest and hesitancies. Vaccine. (2022) 40:4057–63. 10.1016/j.vaccine.2022.05.070 35660035 PMC9149202

[31] Garett R Young SD. Online misinformation and vaccine hesitancy. Transl Behav Med. (2021) 11:2194–9. 10.1093/tbm/ibab128 34529080 PMC8515268

[32] Ashkenazi S Livni G Klein A Kremer N Havlin A Berkowitz O. The relationship between parental source of information and knowledge about measles/measles vaccine and vaccine hesitancy. Vaccine. (2020) 38:7292–8. 10.1016/j.vaccine.2020.09.044 32981777

[33] Tankwanchi AS Bowman B Garrison M Larson H Wiysonge CS. Vaccine hesitancy in migrant communities: a rapid review of latest evidence. Curr Opin Immunol. (2021) 71:62–8. 10.1016/j.coi.2021.05.009 34118728

[34] Ewald LPF. Combatting Vaccine Hesitancy Through Storytelling: Four Key Insights on the Power of this Persuasive Approach. (2021). Available online at: https://www.linkedimmunisation.org/blog/combating-vaccine-hesitancy-through-storytelling-four-key-insights-on-the-power-of-this-persuasive-approach/ (accessed October 12, 2021).

[35] Wirtz VJ Millán-Garduño G Hegewisch-Taylor J Dreser A Anaya-Sanchez A González-Vázquez TT. Misinformation messages shared via WhatsApp in Mexico during the COVID-19 pandemic: an exploratory study. Health Promot Int. (2023) 38:daad041. 10.1093/heapro/daad041 37140349

[36] Bowles J Larreguy H Liu S. Countering misinformation via WhatsApp: preliminary evidence from the COVID- 19 pandemic in Zimbabwe. PLoS ONE. (2020) 15:e0240005. 10.1371/journal.pone.0240005 33052967 PMC7556529

[37] Kantor J. The power of narrative: storytelling, fear, and the COVID-19 pandemic. JAAD Int. (2021) 5:9–10. 10.1016/j.jdin.2021.07.005 34746875 PMC8561011

[38] Centers for Disease Control and Prevention Immigrant and Refugee Health: Vaccination 2024. (2024). Available online at: https://www.cdc.gov/immigrant-refugee-health/hcp/domestic-guidance/immunizations.html (accessed June 15, 2024).

[39] Siddiq H Rosenberg J. Clinicians as advocates amid refugee resettlement agency closures. J Public Health Policy. (2021) 42:477–92. 10.1057/s41271-021-00296-9 34290364 PMC8294283

[40] Essex R Kalocsányiová E Rumyantseva N Jameson J. Trust amongst refugees in resettlement settings: a systematic scoping review and thematic analysis of the literature. J Int Migr Integr. (2022) 23:543–68. 10.1007/s12134-021-00850-0

[41] Nageeb S Vu M Malik S Quinn MT Cursio J Padela AI. Adapting a religious health fatalism measure for use in Muslim populations. PLoS ONE. (2018) 13:e0206898. 10.1371/journal.pone.0206898 30388161 PMC6214560

[42] Gele A Sheikh NS Kour P Qureshi SA. Uptake of COVID-19 preventive measures among 10 immigrant ethnic groups in Norway. Front Public Health. (2022) 10:809726. 10.3389/fpubh.2022.809726 35812507 PMC9259830

[43] Ross Perfetti A Abboud S Behme M Barg FK. Understanding wellness and barriers to care among Iraqi refugee women in the United States. Health Soc Care Community. (2019) 27:1430–7. 10.1111/hsc.12810 31338949

[44] Chace Dwyer S Mathur S Kirk K Dadi C Dougherty L. “When you live in good health with your husband, then your children are in good health…” a qualitative exploration of how households make healthcare decisions in Maradi and Zinder Regions, Niger. BMC Public Health. (2022) 22:1350. 10.1186/s12889-022-13683-y 35840957 PMC9283840

[45] Lin Y Cai CZ Hu Z Zimet GD Alias H Wong LP. The influence of men on HPV vaccination of their spouse/partner in China. Hum Vaccin Immunother. (2022) 18:2049132. 10.1080/21645515.2022.2049132 35380926 PMC9196783

