# Supplementary material for: COVID-19 vaccine barriers among pregnant and lactating refugee women: a case study
Source: Front Public Health. 2025 Jul 8;13:1600107. doi: 10.3389/fpubh.2025.1600107 (PMC12279790; doi:10.3389/fpubh.2025.1600107)
Supplement: Supplementary file 1 [file Supplementary_file_1.docx]

Appendix 1: Interview Guide for Vaccine-Hesitant Pregnant/Lactating Refugee Women

**Introduction: Thank you for taking the time to participate in this interview. The information will help us better understand how pregnancy and being a new mother influenced your decisions about getting COVID-19 vaccinations.**

- What comes to mind when you hear “COVID-19” or coronavirus?
- How has COVID-19 impacted your life, family, and your community?
- How did you keep your family safe during this time?
- What made you NOT want to get the COVID-19 vaccine?
- What have you heard about the vaccine that stopped you from getting it?
- What did you consider when thinking about getting the COVID-19 vaccine?
- Provider recommendation?
- Cultural Health Navigator recommendation?
- Videos in your language?
- Social media posts?
- In what way did being pregnant influence your decision not to vaccinate?
- What vaccines are you willing to receive during pregnancy?
  - Flu? TDAP? Any other vaccines?
  - ***If none, what do you think about vaccines?
- When you moved to the United States, you were made to receive certain vaccines. How has that influenced your perception of vaccines?
- What else influences your thoughts about vaccines?
- What, if anything, would have changed your mind about getting a COVID-19 vaccine?
- What else would you like to discuss about getting COVID-19 vaccines?

Appendix 2: Interview guide with CHNs

**Thank you for taking the time to participate in this one-time interview. You have been a valuable member of our research team. The purpose of these interviews with the research team cultural health navigators is to explore what you have learned about COVID-19 vaccine hesitancy while working with your communities.**

- What stories do you remember hearing about COVID-19 vaccines when they first came out, that you

believe influenced your communities’ willingness to vaccinate against COVID-19?

- What were your experiences trying to convince pregnant women to vaccinate against COVID-19?
  - In what way did the age of the patient influence these conversations?
  - In what way did women’s pregnancy or breastfeeding status influence their willingness to

vaccinate?

- What do you believe have been the most significant barriers to COVID-19 vaccination in your community?
- What forces have influenced your community’s perceptions or thoughts about COVID-19 vaccines?
  - Politics
  - Culture
  - Religion
- What were your community’s expectations about getting the COVID-19 vaccines? In what way were people expected to or expected not to get vaccinated against COVID-19??
- What do you remember hearing about COVID-19 vaccines at community gatherings (e.g., church, community events, etc.)?
- What do you believe was the role of the media, including social media, in influencing your community’s

COVID-19 vaccine perceptions?

- In what way do you think this influenced your communities’ decision to vaccinate?
- In what way did fear of vaccines influence your community’s willingness to vaccinate against COVID-19?
- Some of your patients received flu and TDAP vaccines during pregnancy but refused COVID-19 vaccines. Why do you think that is?
- What are some differences between how COVID-19 vaccines are seen here in the US and in your communities’ home countries?
- How would you describe your experience interviewing women who did not want to get the COVID-19 vaccine?
- What additional resources do you believe would have been more helpful in meeting your community's needs during the COVID-19 pandemic?
- What do you think the hospital or clinic could have done differently to meet the needs of your community better so that they could receive COVID-19 vaccines?
- What do you think the local public health department or the state could have done differently to address COVID-19 in your community?
- What else would you like to share about your work on this project?

**Thanks so much for your time and energy on this project. That concludes our interview.**

**Appendix 3:** Focus group guide with CHNs

**Introduction: In this focus group, we wanted to explore your experiences recruiting and interviewing former pregnant and lactating refugee women about their COVID-19 vaccine experiences and perspectives. In this group discussion, the rest of the team hoped to learn more about your experiences working in this study.**

- What influenced your communities’ decisions to receive or avoid COVID-19 vaccines?
- What are your communities’ perceptions about COVID-19 vaccines now (currently)?
- In what ways was it challenging to recruit women into the study? Why do you believe it was hard getting them to participate?
  - What were your experiences recording the interviews?
  - In what way do you think their responses may have been affected by recording the conversations?
- How would you describe your experiences during the interviews, encouraging women to share their stories?
  - In what ways could you tell some women were ready to stop sharing their stories?
  - Did any stories stand out to you?
- How did participants share any other information about COVID-19 vaccination after the interview (after you stopped the recorder)?
- How did vaccine-hesitant women respond to you personally after the interviews?
- Is there anything else you wish you had known or could have helped you better prepare for the interviews?
- Is there anything else you would like to share about this project?

**Thanks so much for your time and energy on this project. That concludes our focus group.**

**Appendix 4:** Demographic characteristics of vaccine-hesitant P/L refugee women

|  | **Participants** | **Arabic** | **Burmese** | **Kinyarwanda** | **Somali** | **Swahili** |
| --- | --- | --- | --- | --- | --- | --- |
| **Age** | Range: | 27-40 | 30-37 | 25 – 43 | 23-40 | 28-41 |
|  | Mean: | 34.7 | 34.0 | 33.0 | 30.7 | 34.5 |
| **Time living in** | Range: | 3-10 | 7-9 | 6-9 | 4.5 – 8 | 10 – 13 |
| **the U.S. in** | Mean: | 6.7 | 8.0 | 7.3 | 6.2 | 11.5 |
| **years** |  |  |  |  |  |  |
| **Marital** | Single | -- | -- | 1(33.3%) | 2(66.7%) | -- |
| **Status** | Married | 3(100%) | 3 (100%) | 2 | 1(33.3%) | 3 (100%) |
| **Religion** | Muslim | 3(100%) | 1(33.3%) | -- | 3(100%) | 1(33.3%) |
|  | Christian | -- | 2(66.7%) | 2(66.7%) | -- | 1(33.3%) |
|  | None | -- | -- | 1(33.3%) | -- | 1(33.3%) |
| **Education** | Never |  |  |  |  |  |
|  | attended | -- | -- | 2(66.7%) | 1(33.3%) | 1(33.3%) |
|  | Primary | 1(33.3%) | 1(33.3%) | -- | 1(33.3%) | 1(33.3%) |
|  | Middle | -- | -- | -- | -- | -- |
|  | Some HS | 1(33.3%) | -- | -- | 1(33.3%) | -- |
|  | High School | -- | 2(66.7%) | 1(33.3%) | -- | 1(33.3%) |
|  | College | 1(33.3%) | -- | -- | -- | -- |
| **Employment** | Full-time Part-time Not employed Caretaker of  children | --  --  --  3(100%) | --  --  --  3(100%) | 2(66.7%)  -- 1(33.3%)  -- | -- 1(33.3%)  2(66.7%)  -- | -- 1(33.3%)  --  2(66.7%) |
| **Income** | < $10,000 | -- | -- | -- | 2(66.7%) | 1(33.3%) |
|  | $10,000- | 3(100%) | -- | 1(33.3%) | 1(33.3%) | -- |
|  | $24,999 |  |  |  |  |  |
|  | $25,000- | -- | 3(100%) | 2(66.7%) | -- | -- |
|  | 49,999 |  |  |  |  |  |
|  | No Answer | -- | -- | -- | -- | 2(66.7%) |
| **Car Owner** | Yes | 3(100%) | 3(100%) | 3(100%) | 2(66.7%) | 3(100%) |
|  | No | -- | -- | -- | 1(33.3%) | -- |
| **# Children** | 1  2  3  4  5 | --  1(33.3%)  --  1(33.3%)  1(33.3%) | --  1(33.3%)  1(33.3%)  --  1(33.3%) | 1(33.3%)  --  --  2(66.7%)  -- | --  1(33.3%)  2(66.7%)  --  -- | --  --  2(66.7%)  1(33.3%)  -- |
| **Previous COVID infection** | Yes  No | --  3(100%) | --  3(100%) | 1(33.3%)  2(66.7%) | 1(33.3%)  2(66.7%) | --  3(100%) |
